# Extract from an Article upon "Some Overlooked Sources of Infection"

**Published:** 1899-06

**Authors:** M. L. Dyer


ARTICLE IV.

### EXTRACT FROM AN ARTICLE UPON "SOME OVERLOOKED SOURCES OF INFECTION."

M. L. DYER.

Uncleanliness in dental surgery need only be mentioned briefly in passing, since all discussion of the subject should be politely suggested, alluding as it does to a profession which consists of scientific men who are already somewhat sensitive upon the question of their relations to the medical profession. It is not my intention, however, to direct attention to this desire on the part of some of these gentlemen to be ranked as specialists with the oculist and the aurist and the gynecologist, but rather to the fact that in dental offices in general, or, to put it more mildly, in a very large proportion of dental offices throughout the country, there is a marked negligence in the proper care and sterilization of instruments and appliances. Numerous instances of infection from this source have been traced, and many cases of necrosis and subperiosteal abscesses, to say nothing of constitutional infection, have been among the deplorable results of unclean dentistry. That such results are inexcusable goes without saying, and the mere invitation to extreme care in their work, given on the part of the medical profession, should be all sufficient to cause dental surgeons everywhere, to adopt thorough and complete aseptie methods for the most trivial operations. If not, then let it be made a penal offense to thrust into the soft and highly absorbent tissues of the human mouth, forceps that have recently been polluted by the festering jaws of the scorbutic, the cankerous or the syphilitic, and only cleaned by a more or less careful wiping with a piece of absorbent cotton. A careful and extensive injury reveals the fact that in the great majority of dental offices in California, except in cases of suspected disease, instruments are seldom surgically cleaned, either before or after operations. No surgeon nowadays would lance a boil, or cut for a sliver, without being sure of his bistoury, and no dental surgeon should pull a tooth or incise a gum without equal care.—*Western Dental Journal.*

